# Consensus statements for evaluation and nonpharmacological Management of Psoriatic Arthritis in UAE

**DOI:** 10.1111/1756-185X.14357

**Published:** 2022-06-09

**Authors:** Khalid A. Alnaqbi, Suad Hannawi, Rajaie Namas, Waleed Alshehhi, Humeira Badsha, Jamal Al‐Saleh

**Affiliations:** ^1^ Department of Rheumatology Tawam Hospital Al Ain UAE; ^2^ College of Medicine and Health Sciences UAE University Al Ain UAE; ^3^ Emirates Health Services (EHS) Dubai UAE; ^4^ Ministry of Health and Prevention Dubai UAE; ^5^ Division of Rheumatology, Department of Internal Medicine Cleveland Clinic Abu Dhabi UAE; ^6^ Medcare Hospital Dubai UAE; ^7^ Dr. Humeira Badsha Medical Center Dubai UAE; ^8^ Dubai Hospital Dubai UAE

**Keywords:** assessment tools, guidelines, nonpharmacological approach, overarching principles, psoriatic arthritis, severity

## Abstract

**Objective:**

Psoriatic arthritis (PsA), a chronic inflammatory arthropathy, is often underdiagnosed in Middle Eastern countries, substantially impacting the treatment of affected individuals. This article aims to highlight current unmet clinical needs and provide consensus recommendations for region‐specific evaluation methods and nonpharmacological therapies in the United Arab Emirates (UAE).

**Method:**

An extensive literature review was conducted, focusing especially on global and regional guidelines for the evaluation and treatment of PsA. These form the basis of the consensus statements formulated. Additionally, an expert panel of key opinion leaders from the UAE reviewed these guidelines and available literature at an advisory board meeting to identify unmet needs, bridge clinical gaps in the UAE, and develop consensus statements for the evaluation and treatment of PsA.

**Result:**

The consensus statements were developed based on overarching principles for the management of PsA, evaluation of patients with PsA, and nonpharmacological approaches for the management of PsA. The overarching principles included adopting a targeted, multidisciplinary approach, along with collaboration between rheumatologists and dermatologists in cases of clinically significant skin involvement. The panel also highlighted the value of composite disease severity measures for characterizing clinical manifestations of PsA. In terms of nonpharmacological management approaches, lifestyle modification (comprising dietary change, exercise, and cessation of smoking) and psychotherapy were recommended.

**Conclusion:**

The consensus statements will aid healthcare professionals in clinical decision‐making in the context of PsA.

## 1 INTRODUCTION

Psoriatic arthritis (PsA), an autoimmune disorder characterized by chronic inflammation of the skin and joints, affects approximately 2%‐3% of the general population.^1^ The global prevalence of PsA varies by geographic region and ranges from 0.001% to 0.42%,^2^, ^3^, ^4^ whereas the prevalence of PsA is 0.01%‐0.3% in Middle Eastern countries.^5^, ^6^ Evidence of nail dystrophy, scalp lesions, intragluteal and/or perianal lesions, involvement of three or more sites, male sex, and family history of PsA^7^, ^8^, ^9^ are risk factors for the development of PsA in patients with psoriasis. Approximately 20% of patients diagnosed with PsA may develop a more aggressive form of arthritis, resulting in joint damage.^4^ Studies have shown that in many patients, PsA may progress to erosive disease in as little as 2 years after onset.^10^

Beyond musculoskeletal and skin manifestations, PsA is associated with comorbidities that contribute to the disease burden substantially. The most frequently associated comorbidities include cardiovascular disease, obesity, type 2 diabetes mellitus, metabolic syndrome, hyperlipidemia, hypertension, nonalcoholic fatty liver disease, hyperuricemia, gout, Crohn disease, and depression.^11^, ^12^, ^13^, ^14^, ^15^ Studies have reported that more than 50% of patients diagnosed with PsA are affected by at least one comorbidity. Comorbidities impact disease activity, physical functioning, and the quality of life of patients with PsA and, therefore, are an important consideration in treatment decision‐making.^16^

A key aspect of PsA treatment is understanding the classification criteria and outcome measures used to assess disease activity. Psoriatic arthritis is different from other forms of chronic inflammatory arthritis in terms of its complex clinical presentation. Therefore, it is important for clinicians and rheumatologists to use appropriate classification criteria in clinical practice to optimize care for patients with PsA. Currently, ClASsification criteria for Psoriatic Arthritis (CASPAR) are widely used for recruitment in randomized clinical trials and longitudinal observational studies, and are validated in primary healthcare settings. However, the criteria require the healthcare practitioner to differentiate inflammatory arthritis from other nonspecific aches and pains in tendons and joints, which would pose a challenge for practitioners other than rheumatologists. For this reason, classification criteria that can better define the inflammatory musculoskeletal disease component are required. Furthermore, there are several validated outcome measures defining low, medium, and high disease activity. However, there is no consensus on the use of any specific outcome measure to assess disease activity and evaluate treatment response in patients with PsA.^17^

Therapeutic decisions in PsA are guided by a patient‐centric approach in collaboration with dermatologists, primarily aimed at addressing disease activity, comorbidities, structural damage, and patient‐reported outcomes.^18^, ^19^ Considering the heterogeneity in the clinical manifestations of PsA, it is important to ensure standardized treatment practices to assist practising physicians; rheumatologists, and dermatologists. Dermatologists and rheumatologists should collaborate and coordinate their efforts to achieve optimal care for patients with PsA. Treatment recommendations developed by members of the European League Against Rheumatism (EULAR) and the Group for Research and Assessment of Psoriasis and Psoriatic Arthritis (GRAPPA) have been widely adopted in clinical practice.^20^, ^21^ Apart from pharmacological therapies, nonpharmacological approaches such as lifestyle modification—including overcoming obesity, smoking cessation, reduction in alcohol intake, and low‐impact physical exercises—are beneficial in the context of PsA.^22^, ^23^, ^24^, ^25^

The objectives of this article are to address the gaps in clinical practice recommendations for the assessment of PsA severity and nonpharmacological therapeutic approaches for the treatment of PsA to assist practising physicians in the United Arab Emirates (UAE).

## 2 MATERIALS AND METHODS

Six experts from the Emirates Society for Rheumatology representing different healthcare sectors of the UAE set up advisory board meetings to develop the consensus guidelines. The panel reviewed international and regional guidelines to determine clinical gaps in the evaluation of patients with PsA, as well as nonpharmacological approaches for the management of PsA. This would facilitate the development of consensus statements positioned around the identified gaps for the UAE.

### 2.1 Targeted literature review

An extensive literature review was conducted considering unmet needs in clinical practice in the UAE. The current international and regional guidelines were reviewed by the panel of experts, and comparisons were made with the American College of Rheumatology/National Psoriasis Foundation Guideline (ACR/NPF) for the Treatment of Psoriatic Arthritis 2018, EULAR 2019, GRAPPA 2015, and the 2014 Saudi Practical Guidelines on the Biologic Treatment of Psoriasis.^20^, ^21^, ^26^, ^27^

Based on a review of international and regional guidelines, consensus statements were developed for the following categories—overarching principles, evaluation of patients with PsA, and management of PsA using nonpharmacological approaches. Additionally, overarching principles from the GRAPPA 2020 treatment recommendations were adapted based on regional and cultural specifications for the UAE.^28^ Key findings from the review were presented to the advisory board as statements from the expert panel. The prime objectives were:

1. To review similarities/differences between various international and regional guidelines for PsA treatment.
2. To identify and discuss gaps and unmet needs in current clinical practice for the evaluation and nonpharmacological management of PsA in the UAE.

The consensus statements were generated following the first advisory board meeting; the statements were authenticated and confirmed during the second advisory board meeting. The final statements formulated were then approved by all the members of the panel and put forth as recommendations.

The consensus statements have been presented in two separate parts. The present article, which is the first part, focuses on overarching principles, evaluation of PsA, and nonpharmacological treatment options for PsA. The second part covers consensus statements related to the pharmacological management of PsA (dosing and administration recommendations, treatment recommendations for PsA domains, and consensus statements on efficacy and safety profiles of nonbiological and biological therapies), monitoring requirements for therapies, and management of comorbidities.

## 3 RESULTS

### 3.1 Overarching principles

Based on current international guidelines, the following principles have been proposed for the management of PsA:

1. For the treatment of PsA, clinicians should adapt to both the treat‐to‐target and multidisciplinary approaches.
2. In patients with active PsA, using the treat‐to‐target strategy is recommended, where treatment should be aimed at reaching the target of remission or, alternatively, low disease activity, by regular assessment of disease activity and appropriate adjustment of therapy.
3. Rheumatologists should primarily care for the musculoskeletal manifestations of patients with PsA.
4. In the presence of clinically significant skin involvement, a rheumatologist and a dermatologist should collaborate in the diagnosis and management.
5. Treatment should aim to offer the best care and must be based on shared decision‐making between the patient and rheumatologist, considering disease factors (activity, previous treatment, structural damage, comorbidities), treatment factors (safety and efficacy), and patient factors (access and preference).

### 3.2 Evaluation of patients with psoriatic arthritis

The 2009 GRAPPA recommendations state that patients can be stratified into “mild,” “moderate,” and “severe” categories for each of the clinical manifestations of PsA (peripheral arthritis, skin disease, spinal disease, enthesitis, and dactylitis).^29^ However, it was understood that patients may present with different levels of disease activity and clinical manifestations, and therefore, the 2015 updated GRAPPA statements removed these rigid categorizations and designed treatment approaches based on the disease activity, prognostic factors, comorbidities, and local access to therapies for the individual domains of PsA, namely peripheral arthritis, axial disease, enthesitis, dactylitis, skin psoriasis, psoriatic nail disease, uveitis, and inflammatory bowel disease.^20^, ^21^

The expert panel acknowledged the value of composite disease severity measures for characterizing the clinical manifestations of PsA. The Psoriatic ArthritiS Disease Activity Score (PASDAS) is a widely adopted weighted index measure that incorporates evaluator and patient assessments of visual analogue scale (VAS) scores, tender and swollen joint counts, dactylitis, enthesitis, health‐related quality of life, and C‐reactive protein levels. The Disease Activity for Psoriatic Arthritis (DAPSA) is a composite activity measure adapted from the disease activity index for the assessment of reactive arthritis (DAREA).^30^ The DAPSA has been clinically validated^31^ and performs well on arthritis domains,^32^, ^33^ but was found to be less powerful than the Composite Psoriatic Disease Activity Index (CPDAI) for the other clinical domains of PsA.^33^, ^34^ The CPDAI is a composite measure that includes assessments for six domains of PsA: peripheral arthritis, functional disability, skin, dactylitis, enthesitis, and spinal manifestations.^35^ Unlike DAPSA, the CPDAI composite measure evaluates the extent of disease activity, as well as the effect of a particular domain on physical function and health‐related quality of life, which includes the mental, emotional, and social functioning domains.^36^ Overall, the PASDAS has been shown to perform better than the DAPSA and CPDAI measures, specifically for estimating high and low disease activity.^33^, ^37^, ^38^ The expert panel urges that the PASDAS scoring assessment should be performed by a trained healthcare professional (trained nurse or rheumatology fellow), because rheumatologists do not routinely use this instrument.

For assessment of peripheral joint involvement, the Psoriatic Arthritis Response Criteria (PsARC) is an easy instrument that can be used in clinical practice. The PsARC evaluates tender and swollen joint scores, and physician's and patient's global assessment of disease activity.^39^ The PsARC was able to distinguish between outcomes in the treated and placebo groups in several trials.^40^, ^41^, ^42^ PsARC is no longer part of the Outcome Measures in Rheumatology Clinical Trials core domain set, but some insurance companies in the UAE mandate it for approval of immunosuppressive therapy.

The Minimal Disease Activity (MDA) scoring instrument is a clinically validated, reliable indicator of the state of disease activity at a given point. The MDA aids in the assessment of the treatment target.^43^, ^44^ The MDA consists of seven outcome measures, including evaluation of tender joints, swollen joints, Psoriasis Area and Severity Index (PASI) or body surface area (BSA) patient pain VAS, Patient Global Assessment, Health Assessment Questionnaire (HAQ), and tender entheseal points. The MDA is achieved when five out of seven criteria are met. The MDA can be widely adopted in the routine rheumatology clinic, owing to the ease of evaluating the individual component measures and the absence of blood tests.^45^ Very low disease activity (VLDA), a modified MDA, has been developed and validated in recent studies. It represents the most stringent target for remission in PsA. The VLDA state is achieved when seven out of seven criteria are met.^46^

The Ankylosing Spondylitis Disease Activity Score (ASDAS) is a recently developed composite disease activity score endorsed by the Assessment of SpondyloArthritis International Society (ASAS). The preferred version selected by the ASAS is the ASDAS‐C‐reactive protein, and the alternative is the ASDAS‐erythrocyte sedimentation rate. The ASDAS score correlated well with disease activity and showed good discriminative power, in terms of both physician and patient global assessments of disease severity.^47^, ^48^ The expert panel recognized the lack of validation of ASDAS in patients with PsA and axial involvement. However, the panel suggests that in such cases, the ASDAS be used.^49^, ^50^

Considering the paucity of information on the diagnostic instruments for the screening of patients with PsA, severity assessment of PsA should be performed on a case‐to‐case basis^26^ and should account for the following factors: involvement of joints and damage based on imaging modalities, loss of physical function, impact on quality of life, and patient‐reported outcomes. Patient‐reported outcomes used for PsA, including the Short Form‐12/36, Health Assessment Questionnaire‐Disability Index (HAQ‐DI), Functional Assessment of Chronic Illness Therapy‐Fatigue (FACIT‐F) scales, are used to capture disease activity, pain, physical function, fatigue, and productivity, among others.^51^

The expert panel acknowledged the pivotal role of rheumatologists in the care of patients with PsA and agreed that, for this reason, stratification of disease severity should primarily be based on rheumatological assessment.^20^ Severe PsA should be established in accordance with the ACR/NPF criteria: poor prognostic factors (erosive disease, dactylitis, elevated levels of inflammatory markers such as erythrocyte sedimentation rate and C‐reactive protein attributable to PsA), long‐term damage that interferes with function (eg joint deformities), and highly active disease that causes major impairment to quality of life and rapidly progressive disease.^26^

The important disease activity measures routinely used in clinical practice are provided in Table 1, along with their respective components. Consensus statements on assessing PsA disease severity are presented in Table 2.

**TABLE 1:** Components in calculation of disease activity measures in PsA^40^, ^52^, ^53^, ^54^, ^55^

Components	DAPSA	CPDAI	PASDAS	MDA	PsARC	ASDAS
Clinical assessment						
Tender joint count	68	68	68	68	68	
Swollen joint count	66	66	66	66	66	
PASI		X	X	X		
Enthesitis (LEI)		X	X			
Dactylitis count		X	X			
VAS physician			X		X	
Physician Global						X
Patient questionnaire						
VAS global	X		X	X	X	X
VAS skin						
VAS joints						
VAS pain				X		
Back pain						X
HAQ		X		X		
DLQI		X				
BASDAI		X				X
ASQoL		X				
SF‐36 PCS			X			
PsAQoL						
ASAS partial remission						X
Laboratory assessment						
CRP	X		X			X
ESR						X

*Note:* Consistent use of scoring method for assessment is important in clinical practice.

Abbreviations: ASDAS, Ankylosing Spondylitis Disease Activity Score; ASQoL, Ankylosing Spondylitis Quality of Life; BASDAI, Bath Ankylosing Spondylitis Disease Activity Index; CPDAI, Composite Psoriatic Disease Activity Index; CRP, C‐reactive protein; DAPSA, Disease Activity index for PSoriatic Arthritis; DAS28, Disease Activity Score 28; DLQI, Dermatology Life Quality Index; ESR, erythrocyte sedimentation rate; LEI, Leeds Enthesitis Index; MDA, minimal disease activity; PASDAS, Psoriatic ArthritiS Disease Activity Score; PASI, Psoriasis Area and Severity Index; PsAQoL, Psoriatic Arthritis‐specific Quality of Life; PsA, psoriatic arthritis; PsARC: Psoriatic Arthritis Response Criteria; SF‐36 PCS, Short Form 36 Physical Component Scale; VAS, visual analogue scale.

**TABLE 2:** Consensus statements on assessing disease activity in PsA

1. Assessment of PsA requires consideration of major disease domains, including peripheral arthritis, axial disease, enthesitis, dactylitis, psoriasis, nail disease, uveitis, and inflammatory bowel disease.
2. Instruments that could be considered for measuring activity in patients with PsA include: PASDAS and DAPSA scores, the PsARC, MDA score, and the ASDAS.
PsARC is an easy instrument that can be considered for assessment of disease activity in patients with PsA in clinical practice. Although PsARC is no longer part of the OMERACT core domain set, some insurance companies mandate it for approval of immunosuppressive therapy.
MDA score can be considered a valid and reliable instrument for the assessment of disease activity state and treatment target in patients with PsA.
The ASDAS score can be considered in the assessment of PsA with axial involvement, despite the lack of validation studies.
A combination of two or three of the most preferred instruments can be used to assess disease activity, and the practitioner should have the option to choose an instrument based on patient characteristics and disease involvement.
Stratification of disease activity should be assessed considering one or more of the following parameters:
Involvement of joints
Damage on imaging modalities
Loss of physical function
Quality of life impact
Patient‐reported outcomes (eg SF‐12/36, HAQ‐DI, FACIT‐F scale)
Axial involvement
For stratification of disease activity of PsA, only rheumatological assessment instruments should be considered.
Severe PsA disease includes the presence of one or more of the following (ACR/NPF):
Poor prognostic factors (erosive disease, dactylitis, extensive skin disease)
Long‐term damage that interferes with function (eg joint deformities)
Highly active disease that causes major impairment to quality of life
Rapidly progressive disease
3. Regular assessment of the following is recommended:
Pain
Functional limitation
Quality of life and
Structural damage (eg X‐ray, ultrasound, MRI)
4. Assessment and timely referral of comorbidities and related conditions, such as metabolic syndrome, obesity, cardiovascular disease, psychiatric disease, fibromyalgia, fatty liver disease, malignancies, chronic infections (eg hepatitis B virus/hepatitis C virus), and bone health, is recommended.

Abbreviations: ACR, American College of Rheumatology; ASDAS, Ankylosing Spondylitis Disease Activity Score; DAPSA, Disease Activity in Psoriatic Arthritis; FACIT‐F, Functional Assessment of Chronic Illness Therapy‐Fatigue; HAQ‐DI, Health Assessment Questionnaire‐Disability Index; MDA, minimal disease activity; MRI, magnetic resonance imaging; NPF, National Psoriasis Foundation; OMERACT, Outcome Measures in Rheumatology Clinical Trials; PASDAS, Psoriatic Disease Activity Score; PsA, psoriatic arthritis; PsARC, Psoriatic Arthritis Response Criteria; QoL, quality of life; SF‐12/36, Short Form‐12/36.

### 3.3 Nonpharmacological therapies

It is known that comorbid medical conditions and lifestyle factors (such as obesity, smoking, alcohol intake) and environmental triggers are risk factors for the development of PsA.^23^, ^56^, ^57^ Patients with obesity and PsA are likely to experience chronic inflammation and have more severe disease activity when compared with patients with a normal body mass index. Obesity is an independent risk factor for PsA, but it is also true that patients with obesity have poorer outcomes and response to pharmacological therapies.^22^, ^58^ Although the evidence is limited to draw definitive conclusions,^59^ weight‐loss interventions can be particularly effective in improving disease activity in this population.^60^, ^61^ These patients may directly benefit from the use of a hypocaloric diet plan, either alone or in combination with aerobic physical exercise.^62^ There is evidence that intermittent fasting, such as the circadian system of fasting observed during Ramadan, is associated with improved disease activity in patients with PsA, regardless of the pharmacological therapy they receive.^63^

In accordance with the recommendations of the ACR/NPF,^26^ the expert panel agreed that any form of physical exercise is preferable to none in patients with active PsA.^25^ Despite limited evidence, physical exercise has been shown to improve cardiorespiratory function and health‐related quality of life in patients with active PsA.^64^ Patients with active PsA may also benefit from the use of nonpharmacological interventions such as physical exercise, occupational therapy, massage therapy, and acupuncture.^65^ The expert panel opined that low‐impact physical exercises, such as tai chi, swimming, and yoga, should be encouraged in patients who cannot tolerate high‐impact exercises such as running.

Despite the fact that there have been few studies examining the effect of smoking on treatment outcomes in PsA patients,^66^ it is well established that smoking is strongly linked to radiographic progression and poor prognosis in rheumatoid arthritis (RA).^67^, ^68^, ^69^, ^70^ Smoking cessation is associated with lower disease activity and improved cardiovascular outcomes in patients with RA.^24^ Therefore, in accordance with ACR/NPF, smoking cessation (cigarettes or tobacco) is recommended in patients with PsA.^26^

A significantly high proportion of patients with PsA report poor quality of life, depressive symptoms, anxiety, mood disturbances, and changes in sleep quality.^71^, ^72^, ^73^ It has been reported that higher disease activity and pain scores are correlated with the presence of a comorbid mental condition.^74^ Psychological interventions, therefore, are an important part of the multidisciplinary care plan for the management of PsA. Although studies are lacking for PsA, psychological interventions such as cognitive behavioral therapy, biofeedback, counseling, mindfulness, relaxation (eg tai chi and yoga), and patient education have been shown to have a positive effect on the physical and psychological distress associated with RA.^75^

Considering the value of these interventions in improving quality of life, which can ultimately have a positive impact on disease outcomes, the expert panel recommends the use of psychotherapy in the routine clinical management of PsA. Consensus recommendations for the use of nonpharmacological therapies for PsA are presented in Table 3.

**TABLE 3:** Consensus recommendations for use of nonpharmacological therapies for psoriatic arthritis (PsA)

Recommendations
Diet
Patients with PsA should be provided dietary counseing
Intermittent fasting can have beneficial effects on PsA disease activity, including PsA‐related disorders, such as enthesitis and dactylitis, regardless of the implicated drug therapy
In patients with overweight and obesity, weight loss should be emphasized
Limited intake of alcohol should be encouraged
Exercise
In patients with PsA, some form or combination of physical therapy, exercise, occupational therapy, acupuncture, and massage therapy should be considered
Low‐impact exercises such as yoga, tai chi, and swimming should be encouraged
High‐impact exercises such as running can be considered in patients who have no contraindication to these exercises
Smoking
Smoking (cigarettes and tobacco) cessation should be emphasized
Psychotherapy
Psychotherapy should be considered for patients with PsA, as depression is prevalent in these patients

## 4 CONCLUSION

The present consensus statements are in agreement with established global guidelines on the different aspects of PsA, especially highlighting the evaluation of PsA and nonpharmacological therapies for PsA. These consensus statements can assist healthcare professionals in the UAE to effectively evaluate and treat patients with PsA.

## AUTHOR CONTRIBUTIONS

KAA had a substantive role in drafting the final manuscript. The authors are fully responsible for all the content and editorial decisions; the authors involved themselves at all stages of manuscript development and approved the final version.

## CONFLICT OF INTEREST

The authors have no conflict of interest related to this work. The consensus guidelines were funded by the Emirates Society for Rheumatology, a nonprofit organization.
